# Pediatric thalassemic patients have higher incidence of asthma: A nationwide population-based retrospective cohort study

**DOI:** 10.1371/journal.pone.0258727

**Published:** 2021-11-04

**Authors:** Hsin-Yi Hsieh, Lin-Chi Huang, Hong-Ren Yu, Kuang-Che Kuo, Wan-Hsuan Chen, Chung-Hao Su, Chuan-Pin Lee, Ko-Jung Chen, Yao-Hsu Yang, Jiunn-Ming Sheen

**Affiliations:** 1 Department of Pediatrics, Kaohsiung Chang Gung Memorial Hospital and Chang Gung University College of Medicine, Kaohsiung, Taiwan; 2 Department of Pediatrics, Chiayi Chang Gung Memorial Hospital and Chang Gung University College of Medicine, Chiayi, Taiwan; 3 Health Information and Epidemiology Laboratory, Chang Gung Memorial Hospital, Chiayi, Taiwan; 4 Department of Traditional Chinese Medicine, Chiayi Chang Gung Memorial Hospital and Chang Gung University College of Medicine, Chiayi, Taiwan; Seoul National University College of Medicine, REPUBLIC OF KOREA

## Abstract

**Introduction:**

Patients with hemoglobinopathies have been reported to have higher rates of pulmonary complications. Few studies have investigated the association between thalassemia and asthma in children.

**Methods:**

We used the data of one million individuals randomly selected from the Registry for Beneficiaries of the National Health Insurance Research Database. One thalassemic child was matched with four control children without thalassemia according to sex, birth year, birth season, prematurity, and previous enteroviral infection.

**Results:**

A total of 800 hundred thalassemic children and 3200 controls were included. Children with thalassemia had higher rates of developing asthma (41.81 vs 25.70 per 1000 person-years, *P* < 0.001) than the non-thalassemia controls with an adjusted hazard ratio of 1.37 (95% confidence interval [CI] = 1.19–1.58). Boys in the thalassemia cohort had a significantly higher adjusted incidence hazard ratio (IRR) of asthma than those in the non-thalassemia cohort (adjusted IRR = 1.45, 95% CI = 1.02–1.73). The risk of atopic and nonatopic asthma was higher in the thalassemia cohort than in the non-thalassemia cohort (IRR = 1.3, 1.61, respectively).

**Conclusions:**

Children with thalassemia were more likely to develop asthma. More attention should be paid to the early diagnosis of asthma and prevention of asthma attacks.

## Introduction

Asthma is a chronic inflammatory disease of the airway, which is one of the leading chronic childhood diseases [1] and a major cause of childhood disability [2]. It affects about one-tenth of children in the United States [3] and one-fifth of school children in Taiwan [4–6].

Hemoglobinopathies are genetic disorders characterized by abnormal, dysfunctional hemoglobin molecules or lower amounts of normal hemoglobin molecules. The most common hemoglobinopathies are sickle cell disease (SCD) and thalassemia [7]. Approximately 35% of children with SCD have lower airway obstruction and 77% airway hyperresponsiveness [8].

Thalassemia is an inherited autosomal recessive disorder caused by decreased or absent production of one or more hemoglobin subunits [9]. Alpha thalassemia is characterized by reduced synthesis of alpha-globin chains, whereas reduced synthesis of beta-globin chains results in beta-thalassemia. Patients with thalassemia account for approximately 5% of the world population [10] and up to 6.2% of population in our country [11–14]. Thalassemia can be classified as a silent carrier, minor trait, intermediate or major [15]. It was reported that one-third of patients with beta-thalassemia intermedia and major had restrictive pulmonary function patterns [16] and half of the patients with thalassemia intermedia had pulmonary hypertension [17].

The incidence of asthma in children with asthma is uncertain. The objective of the present study was to investigate the incidence and determine whether children with thalassemia have a higher potential for asthma than children without thalassemia.

## Methods

### Data source

Taiwan launched a single-payer National Health Insurance Program on March 1, 1995. Approximately 99% of the 23 million Taiwanese citizens were enrolled in the program. The National Health Research Institutes was authorized by the Bureau of National Health Insurance to create the National Health Insurance Research Database (NHIRD) for medical research. This database contains administrative and health claims data. In this study, we used the Longitudinal Health Insurance Database 2010 (LHID2010), which is a subset of the NHIRD comprising patient data from 1996 to 2013. The LHID2010 comprises data on one million beneficiaries randomly sampled from the original NHIRD. This study was approved by the Institutional Review Board of Kaohsiung Chang-Gung Memorial Hospital (Permit No CGMF- 201801200B0), Taiwan. Because this was secondary data analysis, all identifications of patients and institutions in NHIRD were removed before the data release, and the informed consent was not applicable.

### Sampled patients

Persons born between 1997 and 2010 were included in the study cohort. These individuals were then separated into a group with thalassemia (ICD-9-CM code 282.4) and a group without thalassemia. Preterm birth and enteroviral infections have been reported to have a higher potential to develop asthma in the future [18, 19]. Therefore, we matched one thalassemic child with four control children without thalassemia according to gender, year of birth, birth season, prematurity (ICD-9-CM code 765.0) and enterovirus infection according to the propensity score model. The code of prematurity was diagnosed at least once in the outpatient department or upon admission. The propensity score was calculated using the Statistical Analysis System 9.4 program (SAS Institute, Cary, North Carolina, USA). We used the term “HPF infections” to include herpangina (ICD-9-CM code: 074.0); hand-foot-and-mouth disease (HFMD, ICD-9-CM code: 074.3); enteroviral infection (ICD-9-CM code: 008.67); meningitis due to enterovirus, coxsackievirus, and echovirus (ICD-9-CM code: 047, 047.0, and 047.1); other enterovirus diseases of central nervous system (ICD-9-CM code: 048); specific diseases related to coxsackievirus (ICD-9-CM code: 074, 074.1, 074.2, 074.20, 074.21, 074.23 and 074.8); and echovirus and coxsackievirus infection (ICD-9-CM code 079.1 and 079.2).

Patients in both the thalassemia and non-thalassemia cohorts were followed up until they were diagnosed with asthma (ICD-9-CM: 493.xx), or death, or the end of 2013. The index date was defined as the birth date. To improve data accuracy, the selection criteria for thalassemia and atopic dermatitis (AD; ICD-9-CM code 691.xx), allergic rhinitis (AR; ICD-9-CM code 477.xx), and HPF infections required all cases of ICD-9 code to be diagnosed at least three times in the outpatient department or once at hospitalization in one year. HPF infections were enrolled only before the date of diagnosis of asthma while atopic dermatitis and allergic rhinitis were enrolled regardness of whether they were diagnosed before or after the date of diagnosis of asthma. The date of access to the database was 2020/04/21.

### Statistical analysis

We used the Chi-square test and Mann-Whitney U test to compare the distributions of categorical demographics and clinical characteristics between the thalassemia and non-thalassemia cohorts. The incidence rate ratios (IRRs) and 95% confidence intervals (CIs) were estimated using Poisson regression. Univariate and multivariate Cox proportional hazard regression models were used to estimate hazard ratios (HRs) and 95% CIs for asthma. The Kaplan-Meier method was used to compare the cumulative incidence of asthma between the two cohorts, and the log ranktest was used to examine the differences. All statistical analyses were performed using SAS software, version 9.3 (SAS Institute, Cary, NC, USA). *P*-value < 0.05 in 2-tailed tests was considered statistically significant.

## Results

**Fig 1:**
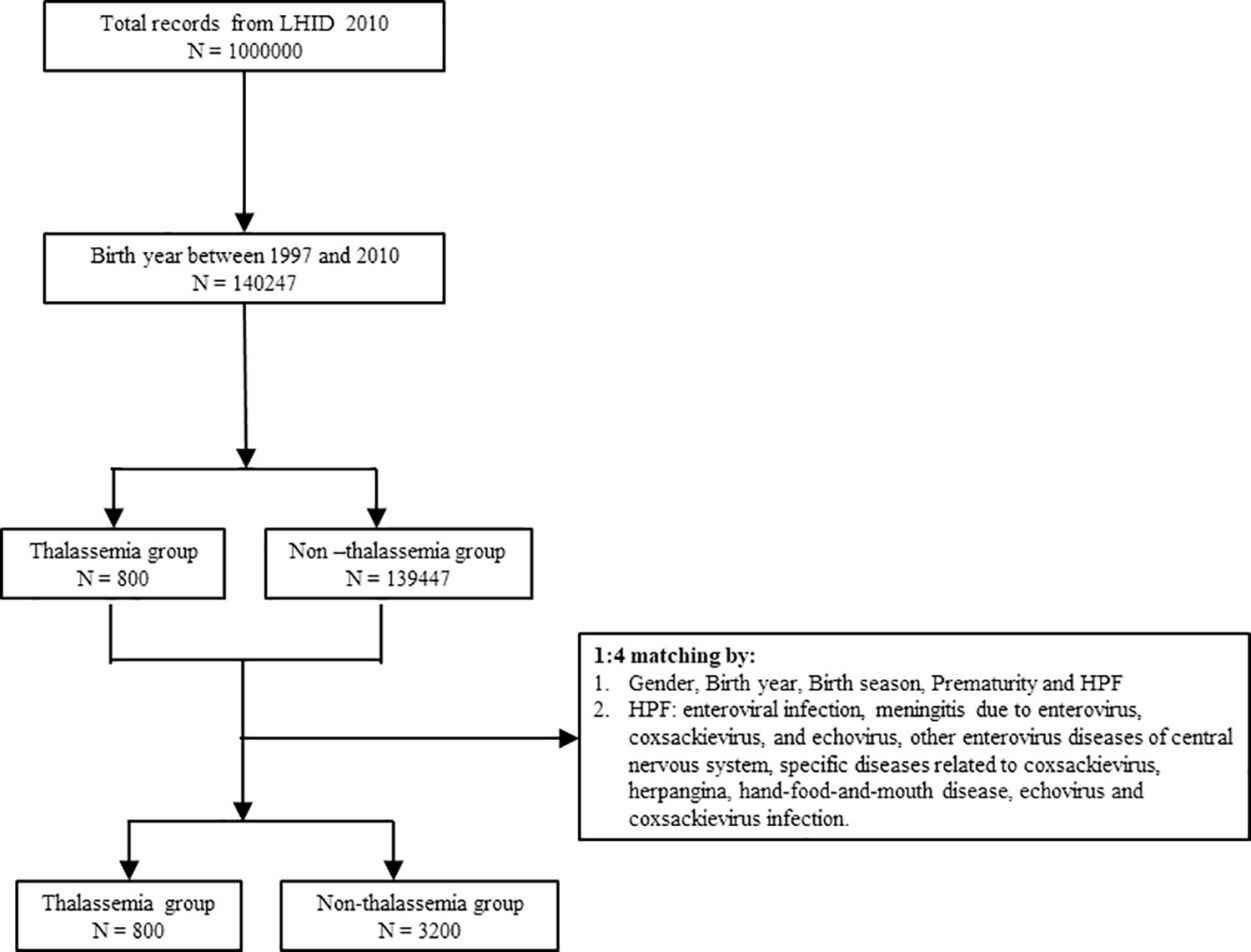
Flow chart of matched cohorts selection. One million people were randomly selected from the Longitudinal Health Insurance Database 2010 (LHID 2010). After the screening process, 800 persons in the thalassemia group and 3200 persons in the non-thalassemia group were analyzed.

One million people were randomly selected from LHID 2010. There were 140247 persons whose years of birth were between 1997 and 2010 selected for further analysis. Next, 140247 children were separated into a group with thalassemia and a group without thalassemia, with 1:4 matching according to gender, birth year, the season of birth, prematurity and HPF infection according to the propensity score model. The details of these processes are shown in Fig 1. Finally, 800 patients were identified in the thalassemia group and 3200 controls in the non-thalassemia group.

Table 1 shows the demographic characteristics of the children with and without thalassemia. In total, 23.4% of children had asthma. Thalassemic children had a higher incidence of asthma than children without thalassemia (33.6% vs. 23.2%, *P* < 0.001). The age at diagnosis of asthma was lower in the thalassemia group than the non-thalassemia group (3.52 vs. 3.97 years old, *P* < 0.001).

**Table 1:** Demographic characteristics of children with thalassemia versus without thalassemia.

	Cohort							Matched cohort				
	Total (n = 140247)		THA (n = 800)		Non-THA (n = 139447)			THA (n = 800)		Non-THA (n = 3200)		
	N	%	N	%	N	%	*P*	N	%	N	%	*P*
Gender							< 0.001					1.000
Girl	66817	47.6	331	41.4	66486	47.7		331	41.4	1324	41.4	
Boy	73430	52.4	469	58.6	72961	52.3		469	58.6	1876	58.6	
Birth year							0.476					1.000
1997–2000	49962	35.6	274	34.3	49688	35.6		274	34.3	1096	34.3	
2001–2005	49469	35.3	278	34.8	49191	35.3		278	34.8	1112	34.8	
2006–2010	40816	29.1	248	31	40568	29.1		248	31	992	31	
Birth season							0.086					1.000
Spring	33926	24.2	209	26.1	33717	24.2		209	26.1	836	26.1	
Summer	34606	24.7	210	26.3	34396	24.7		210	26.3	840	26.3	
Autumn	36837	26.2	212	26.5	36625	26.2		212	26.5	848	26.5	
Winter	34878	24.9	169	21.1	34709	24.9		169	21.1	676	21.1	
Prematurity							0.041					1.000
yes	3647	2.6	30	3.8	3617	2.6		30	3.8	120	3.8	
no	136600	97.4	770	96.2	135830	97.4		770	96.2	3080	96.2	
HPF							< 0.001					1.000
yes	31200	22.2	307	38.4	30893	22.2		307	38.4	1228	38.4	
no	109047	77.8	493	61.6	108554	77.8		493	61.6	1972	61.6	
PS* (×100)	0.57	0.24	0.67	0.29	0.57	0.24		0.67	0.29	0.67	0.29	
Mean (SD)												
Median (Q1-Q3)	0.48	0.41–0.57	0.55	0.44–0.95	0.48	0.41–0.57		0.55	0.44–0.95	0.55	0.44–0.95	
Asthma	32457	23.1	269	33.6	32188	23.1	< 0.001	269	33.6	742	23.2	< 0.001
Age at diagnosis							0.009					0.004
Mean (SD)	3.89	2.37	3.52	2.19	3.90	2.37		3.52	2.19	3.97	2.33	
< 1y/o	2791	2.0	34	4.3	2757	2.0		34	4.3	58	1.8	
1-3y/o	9862	7.0	92	11.5	9770	7.0		92	11.5	223	7	
3-6y/o	15012	10.7	114	14.2	14898	10.7		114	14.2	349	10.9	
> 6y/o	4792	3.4	29	3.6	4763	3.4		29	3.6	112	3.5	

HPF, any morbidity of herpangina, hand-food-and-mouth disease, enteroviral infection, meningitis due to enterovirus, coxsackievirus, other enterovirus diseases of central nervous system, echovirus and coxsackievirus infection; THA, thalassemia; PS*, Propensity score estimated by logistic regression model with THA as dependent variable and gender, birth year, birth season, prematurity and HPF as independent baseline variables for cohort and matched cohort.

Table 2 shows the IRRs of asthma in children with thalassemia versus those without thalassemia. The overall incidence of asthma was 63% higher in the thalassemia cohort than in the non-thalassemia cohort (41.81 vs 25.70 per 1000 person-years), with an adjusted IRR (aIRR) of 1.37 (95% CI = 1.19–1.58). In an analysis in which the patients were stratified according to sex, boys in the thalassemia cohort had a significantly higher aIRR of asthma than those in the non-thalassemia cohort (aIRR = 1.45, 95% CI = 1.22–1.73). In patients whose birth years were 1997–2000 and 2001–2005, the risk of asthma was 1.51- and 1.44-fold higher in the thalassemia cohort than in the non-thalassemia cohort. About the season of birth, the risk of asthma was 1.46, 1.50-fold higher, respectively, in patients who were born in spring and autumn in the thalassemia cohort. In patients who were born prematurely or not, the risk of asthma was higher in the thalassemia cohort (aIRR = 1.88, 1.35, respectively). With regard to HPF infection, the risk of asthma was also higher in the thalassemia cohort (aIRR = 1.36, 1.37, respectively). Next, we analyzed the AD and AR subgroups. Thalassemic children had a higher rate of atopic asthma (with AD or AR) and non-atopic asthma (neither AR nor AD) than the non-thalassemia cohort (aIRR = 1.3, 1.61, respectively).

**Table 2:** Incidence rate ratio of asthma in children with thalassemia versus without thalassemia.

		THA (n = 800)					Non-THA (n = 3200)									
	N	Times	PY	Rate (per 1000 PY)^a^		N	Times	PY	Rate (per 1000 PY)		IRR (95% CI)		*P*	Adjusted IRR (95% CI)		*P*
Overall	800	269	6433.15	41.81	(37.40-47.12)	3200	742	28872.52	25.7	(23.92-27.62)	1.63	(1.42-1.87)	<0.001	1.37	(1.19-1.58)	<0.001
Girl	331	92	2884.88	31.89	(26.00-39.12)	1324	255	12367.78	20.62	(18.24-23.31)	1.55	(1.22-1.96)	<0.001	1.25	(0.98-1.59)	0.075
Boy	469	177	3548.27	49.88	(43.05-57.80)	1876	487	16504.84	29.51	(27.00-32.25)	1.69	(1.42-2.01)	<0.001	1.45	(1.22-1.73)	<0.001
Birth year																
1997-2000	274	101	2993.66	33.74	(27.76-41.00)	1096	262	13714.22	19.1	(16.93-21.56)	1.77	(1.40-2.22)	<0.001	1.51	(1.20-1.90)	0.001
2001-2005	278	105	2183.42	48.09	(39.72-58.23)	1112	273	9997.03	27.31	(24.25-30.75)	1.76	(1.41-2.21)	<0.001	1.44	(1.14-1.80)	0.002
2006-2010	248	63	1256.08	50.16	(39.18-64.21)	992	207	5161.26	40.11	(35.00-45.96)	1.25	(0.94-1.66)	0.12	1.13	(0.85-1.50)	0.39
Birth season																
Spring	209	72	1,678.66	42.89	(34.05–54.04)	836	194	7,661.76	25.32	(22.00–29.15)	1.69	(1.29–2.22)	<0.001	1.46	(1.11–1.92)	0.006
Summer	210	72	1,714.60	41.99	(33.33–52.90)	840	205	7,526.43	27.24	(23.75–31.23)	1.54	(1.18–2.02)	0.002	1.25	(0.95–1.64)	0.112
Autumn	212	75	1,588.83	47.2	(37.64–59.19)	848	189	7,317.71	25.83	(22.40–29.79)	1.83	(1.40–2.39)	<0.001	1.5	(1.15–1.97)	0.003
Winter	169	50	1,451.06	34.46	(26.12–45.46)	676	154	6,366.62	24.19	(20.65–28.33)	1.42	(1.04–1.96)	0.03	1.28	(0.93–1.77)	0.128
Prematurity																
yes	30	16	219.05	73.04	(44.75–19.23)	120	40	1,034.03	38.68	(28.38–52.74)	1.89	(1.06–3.37)	0.032	1.88	(1.05–3.39)	0.034
no	770	253	6,214.10	40.71	(35.99–46.05)	3,080	702	27,838.49	25.22	(23.42–27.15)	1.61	(1.40–1.86)	<0.001	1.35	(1.17–1.56)	< 0.001
HPF																
yes	307	88	2509.04	35.07	(28.46–43.22)	1228	247	10899.89	22.66	(20.00–25.67)	1.55	(1.21–1.97)	<0.001	1.36	(1.07–1.74)	0.013
no	493	181	3,924.11	46.13	(39.87–53.36)	1972	495	17,972.63	27.54	(25.22–30.08)	1.67	(1.41–1.99)	<0.001	1.37	(1.15–1.62)	< 0.001
AD/AR																
with AD	193	75	1,296.80	57.83	(46.12–72.52)	515	184	3,737.36	49.23	(42.61–56.89)	1.17	(0.90–1.54)	0.24	1.17	(0.89–1.53)	0.267
w/o AD	607	194	5,136.36	37.77	(32.81–43.48)	2,685	558	25,135.16	22.2	(20.43–24.12)	1.7	(1.44–2.00)	<0.001	1.45	(1.23–1.71)	< 0.001
with AR	412	200	2,987.19	66.95	(58.29–76.91)	1,245	510	10,135.81	50.32	(46.13–54.88)	1.33	(1.13–1.57)	0.001	1.31	(1.11–1.54)	0.001
w/o AR	388	69	3,445.97	20.02	(15.81–25.35)	1,955	232	18,736.71	12.38	(10.89–14.08)	1.62	(1.24–2.12)	0.001	1.53	(1.16–2.00)	0.002
with AD or AR	493	215	3,593.00	59.84	(52.35–68.40)	1,455	544	11,794.70	46.12	(42.41–50.17)	1.3	(1.11–1.52)	0.001	1.3	(1.11–1.52)	0.001
with AD and AR	112	60	690.98	86.83	(67.42–11.83)	305	150	2,078.47	72.17	(61.50–84.69)	1.2	(0.89–1.62)	0.226	1.18	(0.87–1.59)	0.295
with AD w/o AR	81	15	605.82	24.76	(14.93–41.07)	210	34	1,658.89	20.5	(14.64–28.68)	1.21	(0.66–2.22)	0.542	1.25	(0.67–2.34)	0.477
with AR w/o AD	300	140	2,296.21	60.97	(51.66–71.95)	940	360	8,057.34	44.68	(40.29–49.54)	1.36	(1.12–1.66)	0.002	1.38	(1.13–1.68)	0.001
w/o AD w/o AR	307	54	2,840.15	19.01	(14.56–24.83)	1,745	198	17,077.82	11.59	(10.09–13.33)	1.64	(1.21–2.22)	0.001	1.61	(1.19–2.18)	0.002

^a^ Rate, incidence rate, per 1000 person years; AD, atopic dermatitis; AR, allergic rhinitis; IRR, incidence rate ratio; PY, person-year.

**Fig 2:**
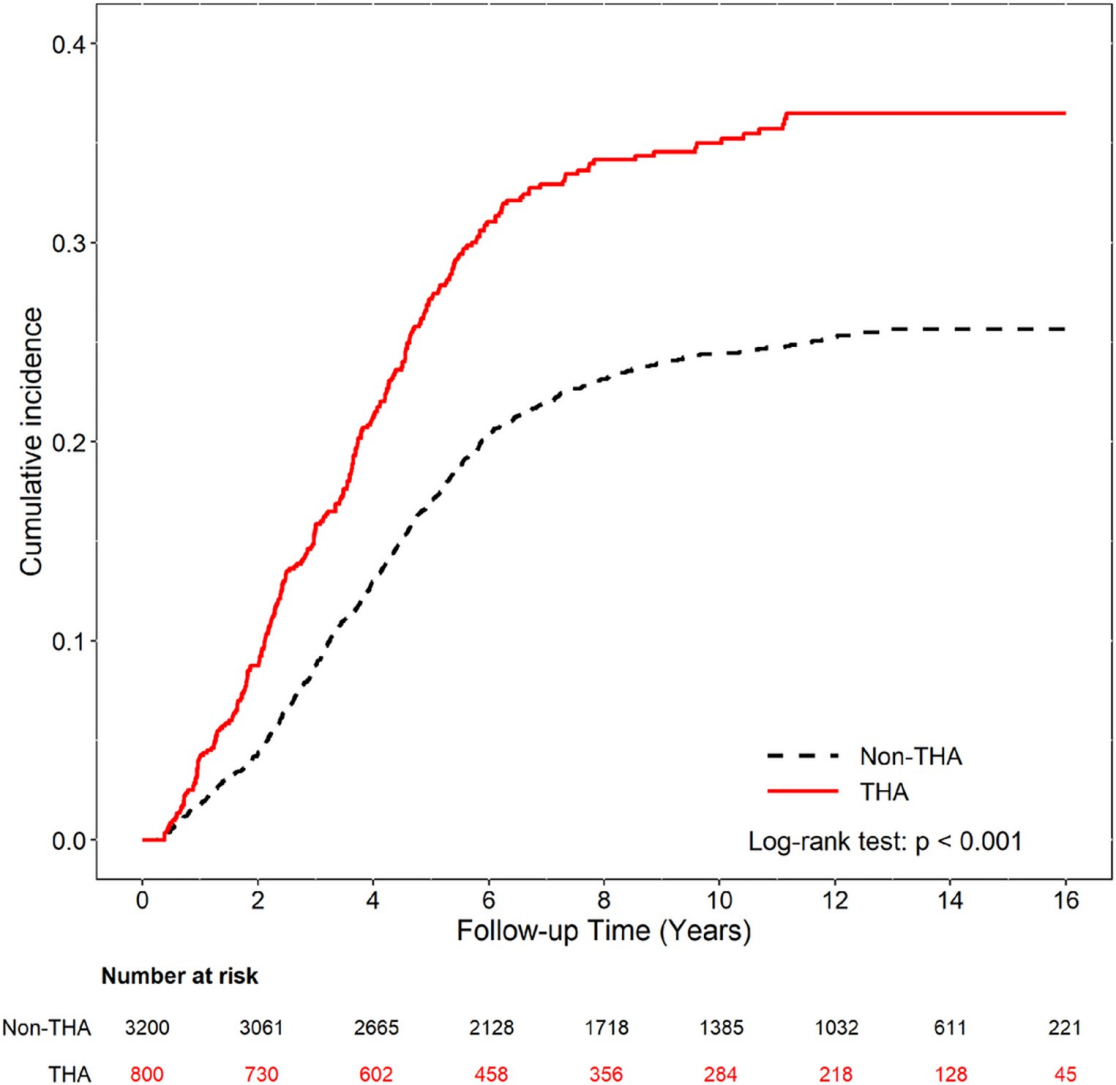
Cumulative incidence comparison of asthma for children with (solid line) or without (dashed line) thalassemia disease.

Next, we analyzed the HR of thalassemia in non-thalassemia children with asthma. Cox regression showed results similar to those shown in Table 2. The overall adjusted HR (aHR) for asthma was 1.36 in the thalassemia cohort than in the non-thalassemia cohort (CI = 1.18–1.56). Thalassemic children had a higher incidence of asthma than children without thalassemia in male patients (aHR: 1.42), year of birth 1997–2000 and 2001–2005 (aHR: 1.47, 1.43 respectively), the birth season of spring and autumn (aHR: 1.44, 1.47, respectively), with or without prematurity (aHR: 1.83, 1.34, respectively), with or without previous HPF infection (aHR: 1.37, 1.34 respectively), without atopic dermatitis (aHR: 1.43), with or without allergic rhinitis (aHR: 1.29, 1.53 respectively) (Table 3). The cumulative incidence of asthma was significantly higher in thalassemic children than in children without thalassemia (*P* < 0.001) (Fig 2).

**Table 3:** Hazard ratio of thalassemia to non-thalassemia children had asthma.

		HR (95% CI)	*P*	Adjusted HR (95% CI))	*P*
Overall		1.59 (1.39–1.83)	< 0.001	1.36 (1.18–1.56)	< 0.001
Gender	Girl	1.55 (1.22–1.97)	< 0.001	1.27 (0.99–1.61)	0.057
	Boy	1.62 (1.37–1.93)	< 0.001	1.42 (1.19–1.68)	< 0.001
Birth year	1997–2000	1.72 (1.36–2.16)	< 0.001	1.47 (1.16–1.85)	< 0.001
	2001–2005	1.73 (1.38–2.17)	< 0.001	1.43 (1.14–1.79)	0.002
	2006–2010	1.26 (0.95–1.68)	0.103	1.15 (0.87–1.53)	0.333
Birth season	Spring	1.64 (1.25–2.15)	< 0.001	1.44 (1.10–1.90)	0.008
	Summer	1.53 (1.17–2.00)	0.002	1.25 (0.95–1.64)	0.112
	Autumn	1.78 (1.36–2.32)	< 0.001	1.47 (1.12–1.93)	0.005
	Winter	1.4 (1.02–1.92)	0.04	1.27 (0.92–1.74)	0.151
Prematurity	Yes	1.85 (1.04–3.30)	0.038	1.83 (1.02–3.29)	0.044
	No	1.58 (1.37–1.83)	< 0.001	1.34 (1.16–1.54)	< 0.001
HPF	Yes	1.54 (1.20–1.96)	< 0.001	1.37 (1.07–1.74)	0.013
	No	1.63 (1.37–1.93)	< 0.001	1.34 (1.13–1.59)	< 0.001
AD	Yes	1.17 (0.89–1.53)	0.261	1.16 (0.89–1.53)	0.276
	No	1.68 (1.42–1.98)	< 0.001	1.43 (1.21–1.69)	< 0.001
AR	Yes	1.31 (1.11–1.54)	0.001	1.29 (1.10–1.52)	0.002
	No	1.59 (1.22–2.08)	< 0.001	1.53 (1.17–2.01)	0.002

Adjusted HR was adjusted by gender, birth year, birth season, prematurity, HPF, AD and AR.

If thalassemia was considered as an independent factor for further analysis, crude and adjusted hazard ratios of factors for asthma proved thalassemia to be a risk factor for asthma (HR: 1.36) besides male sex (HR: 1.27), the birth year between 2006–2010 (HR: 1.31), atopic dermatitis (HR: 1.86), and allergic rhinitis (HR: 3.85) while HPF infections were less risky (HR: 0.65) (Table 4).

**Table 4:** Crude and adjusted hazards ratio of factors for asthma.

		Univariate			Multivariate		
		HR	(95% CI)	*P*	HR	(95% CI)	*P*
THA (non-THA as 1)		1.59	(1.39–1.83)	< 0.001	1.36	(1.18–1.56)	< 0.001
Boy (girl as 1)		1.43	(1.25–1.62)	< 0.001	1.27	(1.12–1.45)	< 0.001
Birth year (1997–2000 as 1)	2001–2005	1.09	(0.94–1.26)	0.25	1.12	(0.96–1.29)	0.143
	2006–2010	1.12	(0.96–1.32)	0.154	1.31	(1.11–1.54)	0.002
Birth season (spring as 1)	summer	1.04	(0.88–1.23)	0.661	1.04	(0.88–1.23)	0.656
	autumn	1	(0.84–1.19)	0.995	1.01	(0.85–1.19)	0.945
	winter	0.93	(0.77–1.11)	0.402	0.91	(0.75–1.09)	0.286
Prematurity (no as 1)		1.65	(1.26–2.16)	< 0.001	1.54	(1.18–2.02)	0.002
HPF (no as 1)		0.77	(0.67–0.87)	< 0.001	0.65	(0.57–0.75)	< 0.001
AD (no as 1)		1.86	(1.61–2.14)	< 0.001	1.41	(1.22–1.63)	< 0.001
AR (no as 1)		3.85	(3.37–4.41)	< 0.001	3.71	(3.23–4.45)	< 0.001

## Discussion

This is a nationwide population-based cohort study based on an extremely large database adjusted for risk factors for asthma. The results suggested that thalassemic children had a higher risk develop asthma than children without thalassemia at a younger age. Thalassemic boys had a higher risk for asthma than non-thalassemic boys. In children, irrespective of premature birth, and previous HPF infection, the risk of asthma was higher in the thalassemia cohort.

Ramakrishnan and Borade [20] reported that anemia is a risk factor for childhood asthma. They found that anemic children were 5.75 times more susceptible to asthmatic attacks than non-anemic children. However, in their study, the percentage of patients with thalassemia was not mentioned. Palma-Carlos et al. [21] collected 4.000 patients in an outpatient allergy clinic over 5 years. Of these, 63 patients had thalassemia and 41/63 (65%) had asthma in comparison to 57% of 491 respiratory allergic patients without thalassemia. However, these data were only collected in allergy clinics, and there were only 63 cases of thalassemia. In our study, a large database demonstrated that thalassemic children have a higher percentage of asthma than non-thalassemic children. Although the total incidence of asthma and the age distribution was consistent with previous studies [4, 6, 22, 23], some cases, especially in younger children might have been incorrectly classified as asthma.

Boys are reported to have an increased risk of asthma compared to girls. The frequency of asthma starts to change from being higher in males to higher in females around puberty [24, 25]. In our study, thalassemic boys had a higher rate of asthma than control cohorts which is consistent with previous reports.

The risk of developing asthma was higher in the thalassemia cohort in patients whose birth year was 1997–2000 and 2001–2005, but not 2006–2010. In this study, we followed up the cases until 2013. The follow-up period was not long enough to be a possible cause. The guidelines for the diagnosis of asthma did not show significant changes between year 1997 and 2010 in our country [4, 26]. Interestingly, the hazard ratio for asthma in the year 2006–2010 was higher than that in the other two periods (Table 4); however, it was in the absence of factor thalassemia.

Respiratory syncytial virus (RSV) and rhinovirus have a well-known link with preschool wheezing and asthma [27, 28]; however, the data on RSV and rhinovirus infection are lacking in our database. Recently, children with enterovirus infection were reported to have a higher incidence of asthma in the future [19]. In our study, thalassemic children with or without previous HPF infection had a higher rate of asthma development than non-thalassemic children. Interestingly, the hazard ratio for asthma showed that children with previous enteroviral infections had a lower risk of asthma (Table 4). Lee et al. reported that herpangina was not associated with the subsequent onset of asthma, while HFMD was a factor in reducing the likelihood of asthma later [29]. There are still some other factors affecting the effects of enterovirus.

The development of AD in infancy and subsequent AR then asthma in later childhood is known as the atopic march [30]. Thalassemic children without AD, with or without AR, had a higher rate of asthma than non-thalassemic children. Interestingly, thalassemic children with AD did not have a higher rate of developing asthma than non-thalassemic children with AD. This is in agreement with a previous report that early-onset early resolving AD does not increase the risk of the development of allergic diseases [31].

It is worth noting that asthma development was associated with an increased risk of subsequent cancer in a recent Korean cohort study [32]. They also reported that patients with nonatopic asthma had a greater risk of overall cancer than those with atopic asthma, suggesting that clinicians should be aware of the higher risk of incident cancer among patients with asthma. Meanwhile, in another Korean nationwide cohort, asthma, especially nonatopic asthma, confers a greater risk of susceptibility to severe acute respiratory syndrome coronavirus 2 infection and severe clinical outcomes of coronavirus disease 2019 [33].

It is very important to avoid immortal time bias in observational studies. Immortal time bias is caused when a cohort study is designed so follow-up includes a period in which participants in the exposed group cannot experience the outcome [34]. Thalassemia is an inherited disease, and the individual index date is the date of birth regardness of the time of thalassemia was diagnosed; therefore, there was no immortal bias in this study.

Regarding the possible mechanism of thalassemia in children with higher risk of asthma, Palma-Carlos et al. [21] stated that the hemorheological changes in thalassemia include greater rigidity of red blood cells in the capillary bed, which can contribute to changes in bronchial circulation and bronchial hyperactivity yet without solid evidence. Recently, we found that thalassemic patients had a higher incidence of low respiratory tract infection [35], which may be linked to a higher incidence of asthma or asthma exacerbation [36, 37].

Our study has a few limitations. First, some confounding factors reported to be associated with asthma could not be assessed and controlled in this study, including a family history of asthma [38] and the delivery method [39]. Second, although we used ICD-9 codes to screen out thalassemic patients, some asymptomatic patients were missed because the physicians did not include thalassemia in their diagnostic lists. Finally, the diagnosis of asthma and enterovirus infection was established by physicians and registered in the NHIRD. The differences in the diagnoses of asthma and enterovirus infection could not be controlled by different healthcare providers. Nevertheless, we included only children who had at least 3 ambulatory claims within 1 year or at least one inpatient claim of either asthma or enterovirus infection to prevent this bias.

The major strength of our study lies in the high number of children selected from a nationwide population-based database that contains data on numerous thalassemia cases. Although the detailed pathophysiology of the relationship between thalassemia and asthma may require further study, we recommend that physicians consider the risk of developing asthma in children with thalassemia.

## Conclusions

In our study, children with thalassemia had a higher risk of asthma than non-thalassemic children. More attention should be paid to early diagnosis and prevention. Further studies should be conducted to obtain more information about its pathophysiology.
